# Virus classification and nomenclature: getting it right

**DOI:** 10.1128/jvi.00034-26

**Published:** 2026-02-19

**Authors:** Stuart G. Siddell, Donald B. Smith

**Affiliations:** 1 School of Cellular and Molecular Medicine, University of Bristol, Bristol, United Kingdom; 2 Nuffield Department of Medicine, University of Oxford 6396, Oxford, United Kingdom; St Jude Children's Research Hospital, Memphis, Tennessee, USA

**Keywords:** virus classification, nomenclature, ICTV

## COMMENTARY

We respectfully suggest that, as a leading journal, the editors of the *Journal of Virology* should support the work of the International Committee on Taxonomy of Viruses (ICTV) by using accurate virus taxonomy. This is important for two reasons. First, virus classification provides a framework within which virologists can study virus evolution. Second, the nomenclature of virus taxa provides a vocabulary that facilitates clear and concise communication. However, several papers published recently in the *Journal of Virology* are likely to confuse readers about the taxonomic status of viruses or about their correct taxonomic names.

The ICTV is the body formally responsible for virus taxonomy, which it achieves through the annual consideration and ratification of taxonomy proposals, as well as by publishing the authoritative source of virus taxonomy, the Master Species List (https://ictv.global/msl). The rules governing the orthography and typography of taxon names can be found in “The International Code of Virus Classification and Nomenclature” (https://ictv.global/about/code). Accordingly, virus species names are written in a binomial format, with the first word (the genus) capitalized, followed by the species epithet, and both words italicized. The names of higher taxa are also italicized.

In contrast to virus taxon names, the common names of viruses are not regulated by the ICTV (1). Virus names are chosen by the virologists who study them, often the person who first described the virus. There is no such thing as an official virus name. However, the ICTV encourages the consistent usage of virus names through its curation of the Virus Metadata Resource (https://ictv.global/vmr), a spreadsheet that associates each virus species with the name(s) of one or more viruses assigned to it. The ICTV also encourages the use of virus names without italics or initial capital letters (except for proper names or genus names, which can be capitalized, or when the virus name occurs at the beginning of a sentence), so as to avoid confusion with taxonomic names.

Some examples of aberrant usage in recent *Journal of Virology* articles are outlined in Table 1. For example, French et al. (2) use the name “*Ferret mastadenovirus*,” which, because it is italicized, gives the impression of being a virus species name, whereas this virus is currently unclassified. To avoid confusion, the virus name should be written as “ferret mastadenovirus.” Similarly, the virus name “*Human rotavirus A*” should be written without italics; this virus belongs to the species *Rotavirus alphagastroenteritidis*, and the virus name “rotavirus A” is recommended in the current VMR. French et al. (2) clearly differentiate animal names from animal species names (for example, common brushtail possum vs *Trichosurus vulpecula*); the same distinction should be applied to virus names and virus species names. Other examples (3–5) include the use of an incorrect species name, a binomial species name made into a tripartite species name, the use of italics in a virus name, and the lack of italics in a taxon name.

**TABLE 1:** Confusing virus species name and virus name usage in recent *Journal of Virology* articles

Article	*Journal of Virology* usage	Source of confusion	Virus name in ICTV VMR	ICTV species/taxon name
(2)	*Ferret mastadenovirus*	Looks like a virus species name	Not listed	Not classified
(2)	*Human rotavirus A*	Looks like a virus species name	rotavirus A	*Rotavirus alphagastroenteritidis*
(5)	rotavirus species A	Nonexistent species name, no italics	rotavirus A	*Rotavirus alphagastroenteritidis*
(5)	Reovirales	Taxon name lacks italics	n/a^*a*^	*Reovirales*
(4)	*B. solanum aureimusivi*	Incorrect species name (genus name abbreviated, species epithet split)	tomato golden mosaic virus	*Begomovirus solanumaureimusivi*
(3)	*Cafeteria roenbergensis* virus	Italics used for part of virus name	Cafeteria roenbergensis virus	*Rheavirus sinusmexicani*

^*a*^ n/a, not applicable.

While individual virologists may choose whether or not to engage with virus taxonomy, we suggest that the *Journal of Virology* (and other virology journals) should accept their responsibilities to the discipline by clearly distinguishing between virus names and virus species names using the orthography and typography stipulated by the ICTV. Articles should also differentiate between official ICTV taxonomic names and proposed taxonomic names that have no taxonomic validity. This could be done by requiring authors to confirm that they have checked their use of taxonomic names and virus names against the current MSL and VMR at the time of submission; this can also be done using the “Find the Species” tool (https://ictv.global/search/find_the_species).
